# High Revision Rates of a Cementless Beta-Titanium Alloy Stem with Contamination-Free Roughened Surface in Primary Total Hip Arthroplasty

**DOI:** 10.3390/jcm9072138

**Published:** 2020-07-07

**Authors:** Sandra Stenicka, Carola Hanreich, Rita Babeluk, Bernd Kubista, Alexander Giurea, Irene Katharina Sigmund, Reinhard Windhager, Rainer Kotz, Richard Lass

**Affiliations:** Department of Orthopedics and Trauma Surgery, Medical University of Vienna, Waehringer Guertel 18-20, 1090 Vienna, Austria; sandra.stenicka@meduniwien.ac.at (S.S.); carola.hanreich@gmail.com (C.H.); rita.babeluk@meduniwien.ac.at (R.B.); bernd.kubista@meduniwien.ac.at (B.K.); alexander.giurea@meduniwien.ac.at (A.G.); irene.sigmund@meduniwien.ac.at (I.K.S.); reinhard.windhager@meduniwien.ac.at (R.W.); rainer.kotz@meduniwien.ac.at (R.K.)

**Keywords:** total hip arthroplasty, cementless stem, contamination-free surface, grit-blasted particles, clinical outcome, radiological analysis

## Abstract

Optimal osseointegration of cementless total hip arthroplasty is essential for high stability and long-term survival. The purpose of this follow-up study was to evaluate the clinical and radiological outcome, the complications, and survival rates of a beta-titanium alloy stem with a specific grit-blasted-free surface. In 192 patients (mean age of 64.4 years), 202 consecutive primary total hip arthroplasties were performed using a cementless Hipstar^®^ stem (Stryker, Duisburg, DE). The Harris Hip Score (HHS) was assessed pre-operatively and post-operatively. Radiolucent lines were evaluated and the implant survival rate was calculated using Kaplan-Meier analysis. The mean follow-up was 7.71 years (range of 5.0–14.0 years). Overall, 15 revisions were performed. Early aseptic stem loosening was observed in six cases (2.97%). Radiolucent-lines adjacent to the stem were detected in 73 cases (83.02%), especially (70.46%) in the Gruen zones 1, 7, 8, and 14. The mean postoperative HHS was 92.65 points (range 42–100). The cumulative survival probability of the stem was 94.4% (95% CI 90.3 to 98.5%). Considering aseptic failure as an endpoint, the cumulative survival rate of the stem was 95.3% (95% CI 0.914 to 0.992) at six years of follow-up. Overall, an inferior mid-term implant survival was observed in comparison to well-established cementless stem designs.

## 1. Introduction

Cementless total hip arthroplasty (THA) is a well-established procedure in orthopedic surgery for treating osteoarthritis (OA) of the hip joint. Extensive variations in shape and various insertion techniques have been developed over the last decades [1,2]. 

Improvements in fixation and osseointegration are of special interest in cementless hip stems to achieve long-term stability and high survival rates. Rotational and axial stability of the implant are the main criteria for primary stability. Hydroxyapatite, porous coatings, grit-blasted surfaces, plasma spraying, and osteoconductive materials like specific titanium alloys have been designed to improve biological integration [1,3,4]. During osseointegration of cementless stems, dynamic bone tissue involves the implant interface. To encourage proper osseointegration, the interface, which is a microscopically amorphous rough structure of approximately 20 to 50 µm, should be nearly filled with regenerated bone [5]. Kahnuja et al. [1] mentioned an optimal pore size between 50 and 400 µm of 30–40% of the stem surface in porous metal-coating procedures to maintain mechanical strength. In grit-blasted surfaces, the use of ceramic or glass particles to roughen the structure of an implant surface is a standardized surface-finishing process [6]. However, remnants of blasting materials were observed on grit-blasted surfaces for cementless fixation [7,8]. A release of surface particles into the joint, which causes third-body wear, were described [4,9].

Another procedure to improve the osseointegration is extensive hydroxyapatite-coating of the stem. It was introduced more than 25 years ago to achieve durable biological fixation in order to maintain physiological periprosthetic bone activity by increased osseointegration and minimized stress shielding [10]. In fact, Reikeras [11] could demonstrate good long-term results of hydroxyapatite-coated stems.

Therefore, in 2003, Stryker^®^ (Duisburg, Germany) created a modified contamination-free grit-blasted surface for the Hipstar^®^ stem using a complex procedure for removing residual blasting media, which should enable better osseointegration. Homogenous roughness (average Ra = 5.6 µm, maximum Rt = 55 µm) is achieved by iron-grit blasting, surface-blow cleaning, acid cleaning, tap and distilled water rinsing, and air-blow drying. The stem is made of titanium-molybdenum-zirconium-iron alloy (TMZF^®^), a beta-titanium alloy, containing no AlO³ [12]. Beta-alloys are best due to a higher strength, superior corrosion resistance, and low elastic modulus [5]. Improved fatigue strength of the TMZF^®^ alloy meant that the design of a hip prosthesis with a smaller neck diameter led to an increased range of motion and, therefore, to a lower risk of impingement, wear, subluxation, and dislocation [13]. In addition, a small rectangular cross-section for optimal endosteal blood circulation was designed to improve osseointegration [6]. Chen et al. [14] showed in a meta-analysis that a femoral component with a tapered geometry is supposed to encourage osseointegration as well. Titanium and beta-titanium alloys have a low electrical conductivity leading to oxide layer formation [5]. Both characteristics, the shape of the stem, and the beta-titanium alloy, combined with the iron-grit blasting surface, should improve the osseointegration and, therefore, the primary stability of the stem. 

The main purpose of this study was to evaluate the descriptive midterm results of the cementless Hipstar^®^ stem, as an update of a previous report published by Lass et al. in 2014 [15]. This evaluates clinical and radiological data focusing on revision rates of this device.

## 2. Material and Methods

### 2.1. Study Design and Patients

We retrospectively evaluated the prospectively collected data of 192 consecutive patients (202 hips, mean age 64.4 years) who underwent uncemented primary total hip arthroplasty (THA) with a contamination-free roughened surface and a rectangular cross-section designed stem called Hipstar^®^ (Stryker, Duisburg, Germany) in a single center study between May 2004 and March 2009. The study received ethical approval of the regional institutional review board (Ref. Nr. 391/2006). Patient characteristics and implant related data are described in Table 1 [15]. Primary osteoarthritis, secondary osteoarthritis, femoral head osteonecrosis, or rheumatoid arthritis were the main reasons for primary THA in our cohort. General exclusion criteria of the study were severe developmental hip dysplasia with fixation disability of the hemispherical acetabular cup, infections, or malignant tumors in the patient history. Ten patients underwent bilateral hip reconstruction using the Hipstar^®^ stem, two in a single session, and the remaining eight had their second hip replacement within one or two years. All operations were performed by experienced orthopaedic surgeons using a lateral-transgluteal approach. Both components of the THA, the acetabular cup, and femoral stem were cementless. The acetabular cup position was aimed at an abduction of 40° ± 10° and an anteversion of 15° ± 10°. The femoral stem position was investigated in postoperative radiographs. Our standard postoperative protocol includes antibiotic prophylaxis for 48 hours and thromboembolic prophylaxis with elastic stocking of the operated leg and low-molecular-weight heparin for six weeks. Patients were instructed to walk with partial weightbearing with the aid of two crutches for six weeks after surgery. Prevention of heterotopic bone formation was achieved by indomethacin or post-operative irradiation [15]. 

**Figure 1:**
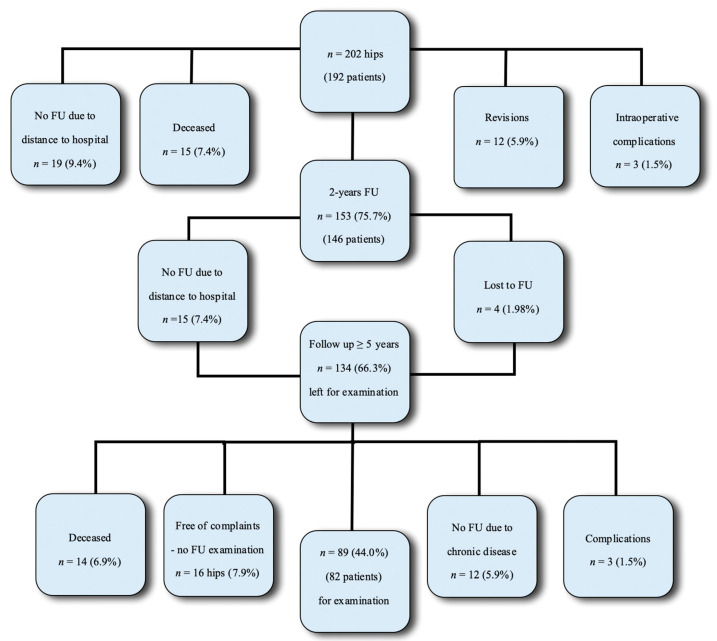
Overview of the follow-up (FU) period.

Of the originally 202 primary total hip arthroplasties in 192 patients, 146 patients (153 cases) were available for the short-term (two years) follow-up examination described by Lass et al. [15] (Figure 1).

Overall, 34 patients could not be included due to the large distance between the hospital and their hometown. Fifteen patients (15 cases) died of other reasons not related to THA within the first two years. Out of the remaining 146 patients (153 cases), seven received a contralateral hip reconstruction. In the meantime, 12 cases were previously excluded in the two years of follow-up because of revisions and, in three cases, intraoperative complications occurred (one periprosthetic fracture, two sciatic nerve lesions) [15].

For the clinical and radiological follow-up, a total of 134 patients were available. Fourteen patients in that group died of causes not related to THA and 12 patients suffered from other chronic diseases leading to an inability to participate in a further follow-up. Sixteen patients were free of complaints and refused to participate in the examinations. 

In the follow-up period, three additional complications (two hip dislocations and one aseptic loosening of the stem) were observed. Overall, 82 patients (89 cases, 41 left and 48 right hips) with a mean follow-up of 7.71 (range 5.0–14.0) years were included in the clinical and radiological examinations including 39 female patients (43 THA) and 36 male patients (39 THA) with an average age of 68.28 (Standard deviation (SD) 2.50) years.

### 2.2. Clinical and Radiographic Evaluation

Standardized clinical and radiographic features were assessed preoperatively and postoperatively at six weeks, three months, six months, one year, and each subsequent year. The clinical evaluation included a physical examination comprising a determination of range of motion and the Harris hip score (HHS). The HHS has a maximum of 100 points and includes pain (44 points), function (47 points), deformity (4 points), and motion (5 points) [16]. 

**Figure 2:**
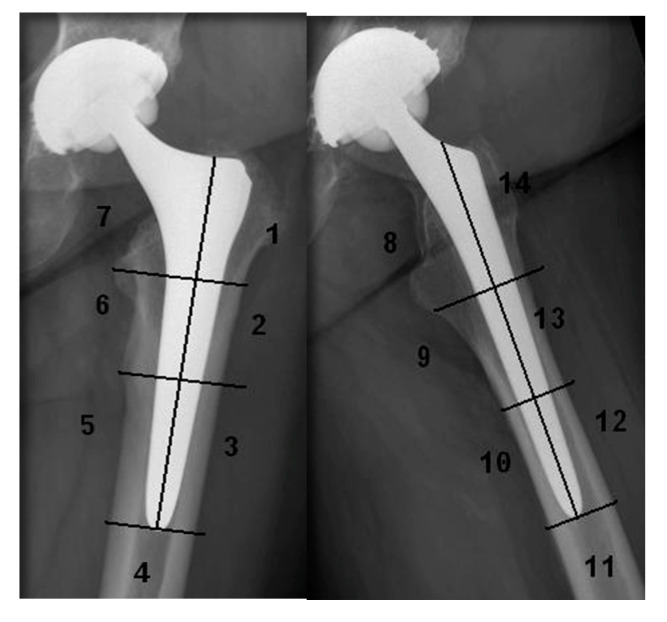
Radiolucent lines classified in Gruen zones (ap. 1–7, lat. 8–14).

Standard radiographs consist of anteroposterior view of the pelvis, and anteroposterior and cross-table lateral views of the hip. A single observer who has not been involved in the operations evaluated all radiographs using a PACS (Patient-Archive-Computer-System) workstation (Agfa HealthCare GmbH, Bonn, Germany). Radiolucent lines of the stem were evaluated, according to the classification system of Gruen et al. [17] on anteroposterior and lateral radiographs (Figure 1). The acetabular components were assessed, according to the system of DeLee and Charnley [18], on anteroposterior radiographs. The radiolucent lines at the bone-prosthesis interface were recorded as <1 mm, between 1 mm and 3 mm, or > 3 mm in width. Radiolucent lines of the cup were recorded as 0 mm, 1 mm, 3 mm, and more than 3 mm in width (Figure 2). 

The osseointegration of the cup was described by Effenberger. Heterotopic ossifications were assessed according to the classification of Brooker et al. [18].

### 2.3. Statistical Analysis

Normally distributed clinical data (Harris hip score, range of motion) were compared using a t-test for paired samples with an alpha level of 0.05. A Kaplan-Meier survival analysis was performed with revision for any reason, revision of the stem for any reason, and for aseptic stem loosening as an endpoint to estimate the implant survival probabilities (revisions as the terminating event and censoring patients at the time of their death or at the end of the follow-up period). All statistical analyses were performed using the Statistical Package for the Social Sciences (SPSS), version 23.0 (SPSS^®^ Inc./IBM, Chicago, Illinois).

## 3. Results

### 3.1. Revisions and Complications

**Figure 3:**
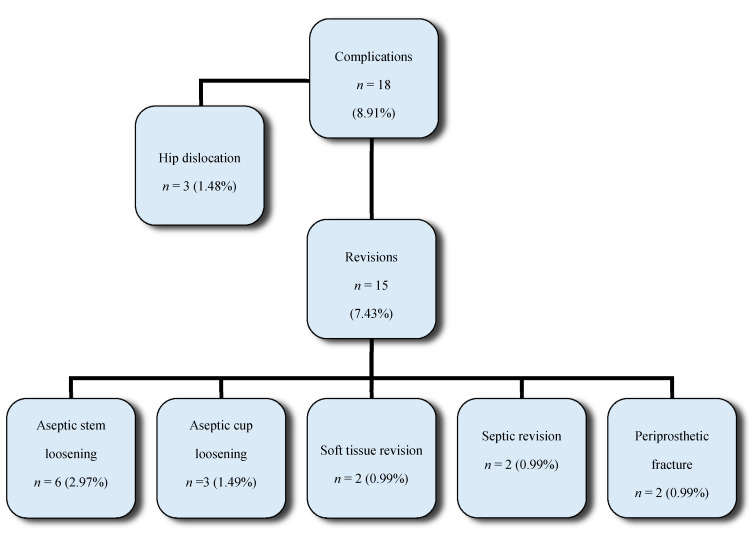
Complications and their required revisions in the follow-up period.

Overall, 18 complications (8.91%) were detected during the observation period. Of these, 15 revisions (7.43%) required a revision surgery (Figure 3).

Three (1.5%) intraoperative complications occurred including one periprosthetic fracture and two reversible sciatic nerve lesions. Twelve additional complications (5.94%) were previously described in the two years of follow-up by Lass et al. [15].

In total, there were six (2.97%) revisions due to aseptic stem loosening at a minimum follow-up of five years. Three cases (1.49%) were observed in the first two years (7, 9, and 18 months, postoperatively), two cases (40 and 48 months, postoperatively) in the fourth year of follow-up. Only one (0.5%) more case occurred in the seventh year (83 months, postoperatively). In three (1.5%) patients, aseptic loosening of the cup was observed within the first two years of follow-up. Four further implant revisions were performed due to two periprosthetic infections and two periprosthetic fractures. One hip dislocation was already reported in our short-term observational study. Furthermore, two patients experienced a dislocation after eight years including one for a traumatic reason in a car accident and one without trauma.

### 3.2. Clinical Results

At a mean follow-up period of 7.71 years (range 5.0–14.0), the mean Harris Hip Score (HHS) of the last clinical examination was 92.7 points (range 42–100) compared to the mean preoperative HHS 47.1 points (range 19–74). Eleven patients showed poor results in the HHS due to other conditions not related to THA. The mean extent of the hip flexion improved from 75° preoperatively to 108° at the last follow-up.

### 3.3. Radiological Results

After excluding the cases of revision, radiolucent lines (RL) were evaluated in radiographs in 89 cases at the last follow-up (mean follow-up of 7.71 years). In three patients, a lateral view was not available. In 73 cases (83.02%), radiolucent lines were detected at the stem. 70.46% of all RL were found in the proximal zones (1, 7, 8, and 14, according to Gruen et al. [17]). More than 50% of all RL were smaller than 1 mm and only 9.25% were larger than 3 mm. Sixteen cases had no RL adjacent to the stem at all (Table 2).

**Figure 4:**
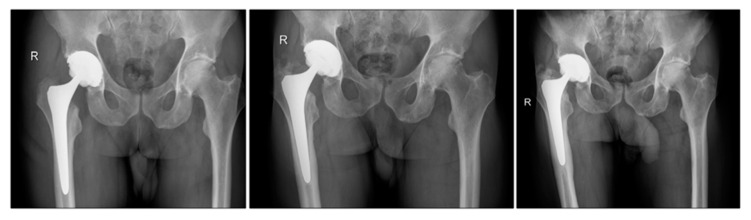
Observed progressive radiolucent lines in anteroposterior view of the pelvic.

Figure 4 shows the loosening process at the stem in one revision case at the first postoperative control, one year postoperative, and four years postoperatively at the time of revision. Radiolucent lines were seen in the proximal zones 1, 7 in the anteroposterior view and 8 and 14 in the lateral view, according to Gruen et al. [17].

### 3.4. Survival Analysis

**Figure 5:**
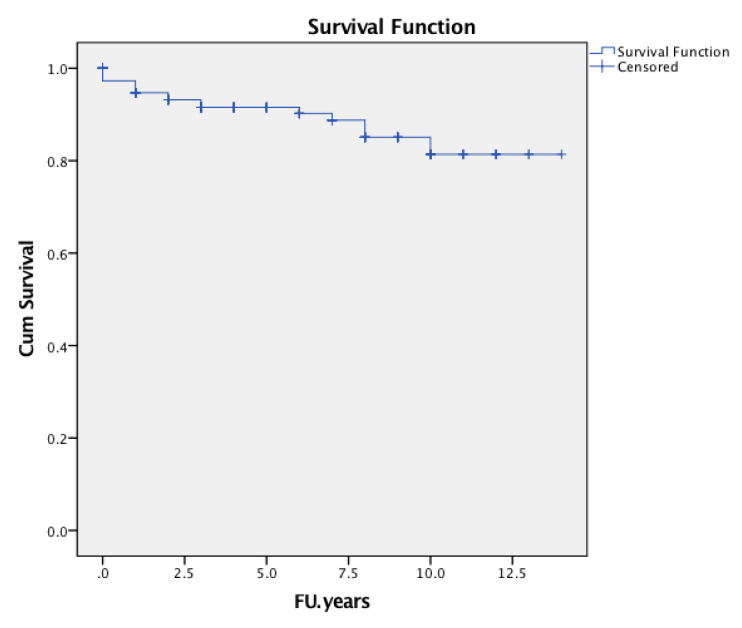
Kaplan-Meier implant survival curve with the endpoint of revision for any reason at the observed period.

**Figure 6:**
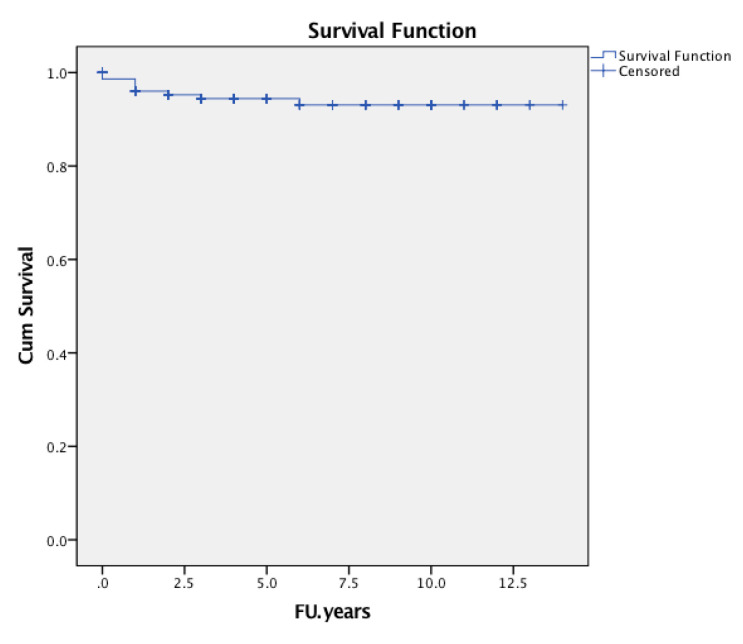
Kaplan-Meier implant survival curve with the endpoint of stem revision for any reason at the observed period.

**Figure 7:**
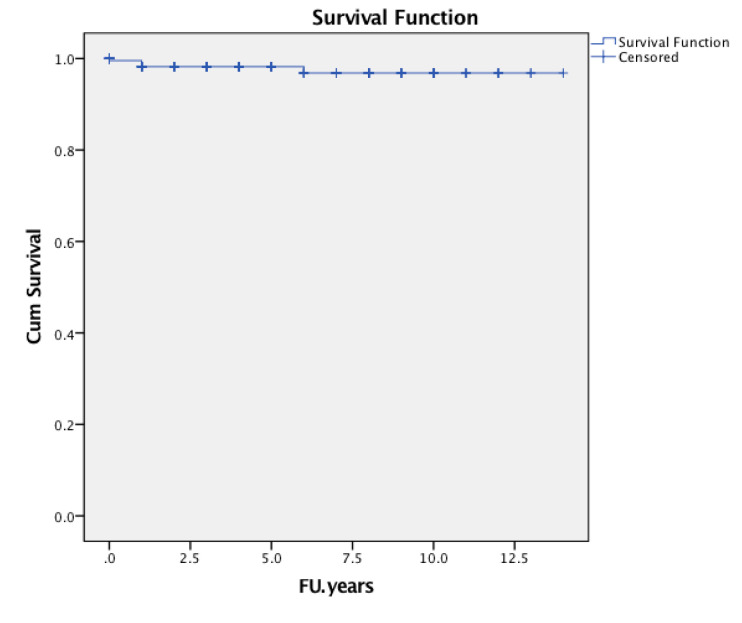
Kaplan-Meier implant survival curve with the endpoint of stem revision for aseptic loosening at the observed period.

The cumulative survival probability of the implant in consideration of any complication was followed by revision of either cup or stem, or both components, as the endpoint was 92.0% (95% confidence interval [CI] 0.873 to 0.967, number of patients at risk during interval t [nt] = 70) (Figure 5). The cumulative survival rate of the stem was 94.4% (95% CI 0.903 to 0.985, nt = 70) considering revisions for any reason (Figure 6), 95.3% (95% CI 0.914 to 0.992, nt = 7) considering revisions for aseptic loosening of the stem as an endpoint at six years of follow-up (Figure 7).

At three years, the cumulative survival probability of the implant was 0.933 (95% CI 0.894 to 0.972, nt = 114), at six years, it was 0.920 (95% CI 0.873 to 0.967, nt = 70) with revision for any reason as the end point. At 7 years, it was 0.905 (95% CI 0.852 to 0.958; nt = 62) and, at 8 years, it was 0.867 (95% CI 0.794 to 0.940; nt = 46) (Figure 5).

At three years, the cumulative survival probability of survival of the stem with revision for any reason as the endpoint was 0.957 (95% CI 0.926 to 0.988, nt = 114), at 6 years, it was 0.944 (95% CI 0.903 to 0.985; nt = 70), and, at 8 years, it was 0.924 (95% CI 0.869 to 0.979, nt = 47) (Figure 6).

At three years, the cumulative survival probability of survival of the stem with aseptic loosening as he endpoint was 0.966 (95% CI 0.937 to 0.955, nt = 114). At 6 years, it was 0.953 (95% CI 0.914 to 0.992, nt = 70) (Figure 7).

## 4. Discussion

Femoral component loosening is the main reason for mechanical failure in total hip arthroplasty. Various proven methods for enhancing osseointegration such as roughening the implant surface or osteoconductive materials like hydroxyapatite coating for titanium alloys were applied for many years [1,3,4,5]. Yet, there are ongoing scientific debates on wear-particle induced loosening in cementless implant fixation. Sharp particles are described to descend from blasting media, such as alumina or glass, which are located from 10% to 20% at roughened surfaces of cementless stems. These particles can migrate into the artificial joint and are a potential reason for early implant loosening [7,8]. Nonetheless, rough surfaces without wear-particles offer the best condition for osseointegration and bone proliferation [7,8]. 

Therefore, the Hipstar^®^ stem was made of titanium-molybdendum-zirconium-iron alloy (TMZF^®^), which is a beta-titanium alloy, without AlO³ for better osseointegration [12]. This homogeneous, rough, contamination-free surface without grit-blasted particles and the small rectangular cross-section improves osseointegration with an optimal endosteal blood circulation, which was previously described by Racey et al. [6]. However, it remains unclear whether the differences in shape or surface are of clinical relevance for avoiding aseptic implant loosening [2]. A recent meta-analysis found out that, in the long-term, hydroxyapatite-coated stems performed better than porous-coated stems with regard to hip scores and survivorship in the long-term [14]. Reikeras [11,19] could demonstrate well-fixed femoral components and good long-term results of hydroxyapatite-coated stems. Hydroxyapatite should also increase the osseointegration and minimize stress shielding, which may cause bone loss in the proximal femoral cortex through adaptive remodeling [10]. The study group described the greatest change in stress distribution at the proximal medial part of the femur. Radiolucent lines adjacent to the stem were found in 36 hips located proximally, mostly lateral of the stem, in a total of 66 hips for 22 years [19]. However, other findings do not support the use of hydroxyapatite coating to enhance implant survival of a femoral stem due to high failures rates [3,11].

Beta-alloy, which is used for the Hipstar^®^ stem surface, leads to an oxide layer formation, which improves the adhesion of osteoblasts improving osseointegration. On the other hand, bioactive coating of the implant surface inhibits fibrous tissue proliferation caused by inflammation and stimulates osteoblast activity. Hydroxyapatite, silicotitanate, or functionalization of implant surfaces with cells, stem cells, or osteoblast cells, can be used for bioactive coating [5,11].

However, even pre-clinical and clinical proofed methods for better osseointegration may contain outliers in implant survival [3,11,15].

A femoral component with a tapered geometry is also supposed to encourage osseointegration [14]. Zweymüller previously designed a similarly structured stem for direct fixation into the metaphysis and diaphysis with a rectangular cross-section design. The average surface roughness was between 3 and 6 µm [20]. In comparison to that stem, the Hipstar^®^ prosthesis has a smaller rectangular cross-section design with a lateral fin to improve primary rotational stability [6,21]. In our short-term follow-up of two years, decreased radiolucent lines (61.4%) compared to other uncemented hip implants with grit-blasted surfaces were found [15,20]. This is consistent with the results of Prymka et al. [22], which shows a significantly higher rotational primary stability of the Hipstar^®^ stem in vitro and fewer radiolucent lines as a result of the rectangular cross-section design. Radiolucent lines are described as a sign of stress shielding, which leads to aseptic loosening, as previously shown in studies of commonly used implants [6,9]. Current studies by Canovas et al. [23,24] paid attention to the shape of press-fit implants for bone fixation. Good clinical outcomes were achieved in cases with proximal press-fit fixation with a smaller extent degree or with short diaphyseal press-fit stems. Therefore, the recommendation is to achieve metaphyseal or short diaphyseal fixation whenever possible. In our early results, radiolucent lines in the proximal zones of the Hipstar^®^ stem were found in up to 61.4%, which are in line with the results by Prymka et al. of 64.04%. This indicates non-metaphyseal primary and secondary anchoring [15,22]. 

In our present mid-term follow-up, the results of the observed radiolucent-lines of the Hipstar^®^ stem (83.02%) are comparable to the results of the well-established Zweymüller stem (SL-Plus^®^) (87%) [20,21]. Several long-term studies related to that stem have failed to confirm that these radiological changes have clinical relevance for aseptic loosening [25,26]. However, in the two years of follow-up of the Hipstar^®^ stem, the numbers of radiolucent lines were remarkably lower (61.4%) [15]. 

At six years of follow-up, the cumulative survival probability of the complete implant (stem and cup) for any reason was 92.0% (95% CI 87.3 to 96.7%). Considering stem revisions for any reason, the cumulative survival probability was 94.4% (95% CI 90.3 to 98.5%) in our follow-up period. The probability of stem survival in consideration of aseptic stem loosening was 95.3% (95% CI 91.4 to 99.2%). Gruebl et al. [27] presented a cumulative survival rate with any revision as an endpoint in 92% (95% CI 88.0 to 97.0%) and with femoral revision as the endpoint in 99% (95% CI 97.0 to 100) at ten years of follow-up and in 98% (95% CI 96.0 to 100%) at 15 years of follow-up for the Zeymüller stem [25]. Kolb et al [28] presented similar results in a follow-up study for the cumulative survival probability of 98% (95% CI 92.0 to 99.0%) with aseptic stem loosening as an endpoint at 20 years. Ateschrang et al. obtained a stem survival rate of 95.0% (95% CI 91.1 to 97.2%) 20 years of follow-up of the Bicontact^®^ stem (B-Braun-Aesculap, Tuttlingen, Germany) [2]. 

In the current study, three revisions due to aseptic stem loosening were performed in the first two years of follow-up, further two in the fourth year, and only one after eight years. However, Kolb et al. reported only two revisions due to aseptic loosening of the Zweymüller stem at 20 years of follow-up (208 total hip arthroplasties) [28].

Our clinical findings of the Hipstar^®^ stem showed better results, mean postoperative HHS of 92.7 points (range 42-100) than recently published investigations of other cementless, total hip stems at similar observation periods. At 10 years of follow-up, Carlson et al. [29] reported a mean HHS of 87.1 points (range 29.7-100) of a summit-triple-tapered stem. Ateschrang et al. obtained a HHS of 81.6 points (SD 18.5) at 20 years of follow-up [2]. The 10 years result of the Zweymüller^®^ stem even showed a lower HHS, 85.4 points (range 46–100 points) compared to the results of the recent study, 92.7 points (range 42–100) [27]. Considering the advanced age of the observed patient group, we would expect a similar development of HHS results. 

The main limitation is the fact that the Hipstar^®^ stem is no longer available on the market since 2011. Another drawback is the variability in radiographic quality and, therefore, the variability in the assessment of stress shielding. Furthermore, associated with increasing age, many patients suffered from comorbidities by possibly interfering with their clinical outcome. All these factors may affect the outcomes adversely.

## 5. Conclusions

The core finding of our follow-up study is the remarkable high rate of revisions, 2.97% (6 cases), caused by aseptic stem loosening. Furthermore, a high incidence of radiolucent lines in up to 83.02% (73 cases), especially in the proximal zones (1, 7, 8, and 14 according to Gruen et al. [17]) of the Hipstar^®^ stem were found in up to 70.46% in our observed period, which indicated a non-metaphyseal primary and secondary anchoring. Despite the modern design and the modified contamination-free roughened surface, this implant showed an inferior outcome when compared with other well-established cementless implant systems.

Even though the stem is not available on the market anymore, the findings of this study are of importance for orthopedic surgeons who have to perform regular follow-ups on the already implanted devices in more than 8900 patients worldwide. Further studies are necessary to describe the radiological and clinical effects of the modified surface in the long-term.

## Figures and Tables

**Table 1:** Demographic and implant-related data [15].

Demographic and Implant-Related Data			
		Mean (Range)	Number (%)
**Number of cases/patients**			202/192
**Age** (years)		64.4 (20 to 89)	
**Weight** (kg)		76 (72 to 80)	
**Sex**	m		87 (43%)
	w		115 (57%)
**Stem size**	0		0.5%
	1		4.7%
	2		14.6%
	3		19.3%
	4		19.3%
	5		19.8%
	6		10.4%
	7		5.7%
	8		5.7%
**Head size**	28		6.8%
	32		56.8%
	36		36.5%
**Cup size**	48		14%
	50		20%
	52		10%
	54		20%
	56		14%
	58		14%
	60		4%
	61		2%
	62		2%

**Table 2:** Distribution of radiolucent lines of the stem.

Radiolucent Lines-Stem [17]				
	Cases with Radiolucent Lines	<1 mm	1–3 mm	>3 mm
**S1**	64.04%	56.14%	35.08%	8.77%
**S2**	20.22%	61.11%	27.77%	11.11%
**S3**	2.25%	0%	100%	0%
**S4**	1.12%	100%	0%	0%
**S5**	3.37%	100%	0%	0%
**S6**	20.22%	66.66%	33.33%	0%
**S7**	46.06%	39.02%	51.21%	9.76%
**S8**	58.13%	52%	40%	8%
**S9**	26.74%	39.13%	43.47%	17.39%
**S10**	1.16%	100%	0%	0%
**S11**	1.16%	100%	0%	0%
**S12**	1.16%	0%	100%	0%
**S13**	17.44%	46.66%	26.66%	26.66%
**S14**	58.13%	52%	42%	16%
